# On designing a configurable UAV autopilot for unmanned quadrotors

**DOI:** 10.3389/fnbot.2024.1363366

**Published:** 2024-05-30

**Authors:** Ali Bhar, Mounir Sayadi

**Affiliations:** ^1^University of Tunis, ENSIT, Labo SIME, Tunis, Tunisia; ^2^Military Academy of Fondouk Jedid, Nabeul, Tunisia

**Keywords:** unmanned aerial vehicles, autopilot, design, PD/PID control, quadrotor

## Abstract

Unmanned Aerial Vehicles (UAVs) and quadrotors are being used in an increasing number of applications. The detection and management of forest fires is continually improved by the incorporation of new economical technologies in order to prevent ecological degradation and disasters. Using an inner-outer loop design, this paper discusses an attitude and altitude controller for a quadrotor. As a highly nonlinear system, quadrotor dynamics can be simplified by assuming several assumptions. Quadrotor autopilot is developed using nonlinear feedback linearization technique, LQR, SMC, PD, and PID controllers. Often, these approaches are used to improve control and to reject disturbances. PD-PID controllers are also deployed in the tracking and surveillance of smoke or fire by intelligent algorithms. In this paper, the efficiency using a combined PD-PID controllers with adjustable parameters have been studied. The performance was assessed by simulation using matlab Simulink. The computational study conducted to assess the proposed approach showed that the PD-PID combination presented in this paper yields promising outcomes.

## 1 Introduction

Recently, there is increasing interest in unmanned aerial vehicles (UAVs) in a wide variety of fields. Recent investigations and researches are published (Akhloufi et al., 2021) regularly since many years. Using UAVs to detect and monitor fire hazards demonstrates an effective contribution, whether they are controlled remotely or incorporated with intelligent computer vision software (Zhang et al., 2014). Indeed, the autonomous drones are becoming an essential tool in fire and rescue management (Moumgiakmas et al., 2021). Even though many technology advances have been made in relevant designing technologies looking for the maximum of efficiency with popular items still challenging. In fact, a huge advancement is continuously made in (MEMS) Micro-Electro-Mechanical Systems and (NEMS) Nano-Electro-Mechanical Systems technologies allowing manufacturing miniature sensors, actuators and controllers (Burggräf et al., 2019). Autonomous applications have to be built for specific missions. Quadrotors are one of the most popular (Manoj Kumar et al., 2021) and their advantages of hovering capabilities, vertical taking off and landing (VTOL) are applied to fixed wing.

The flight behavior of a quadrotor is impacted by several factors such as the wind, the weight variation, the speed, the trajectory to follow and the evolution of its own internal parameters (e.g., Wei et al., 2023). Due to sophisticated nonlinearity and strong coupling parameters under-actuating an absolutely unstable dynamic model, these phenomena are augmented. Indeed, to control a quadcopter we have four fixed rotors to control its six degrees of freedom in space. Any control strategy for manual, or autonomous missions depends on the reliability of the mathematical model. A valid and accurate mathematical model can be extracted from a commercial or self-designed Quadrotor using identification and laboratory experiments (Tahir et al., 2023).

Research has been conducted on the altitude and attitude control methods for quadrotors in the literature. The control strategy including Sliding Mode Control (SMC) is often used (Rinaldi et al., 2023). It presents good robustness results for nonlinear systems in presence of parameter uncertainties and external disturbances (Manoj Kumar et al., 2021). It has been shown that this technique can be applied in model-free control (Wang et al., 2016) or associated with LQR optimization, adaptive or back-stepping modes (e.g., Bouabdallah and Siegwart, 2005; Li et al., 2014; Ghamry et al., 2016) for indoor and outdoor operations. The augmented SMC controller reduces the disadvantage of the chattering problem. There may be undesirable oscillations in practice, called chattering problems, of finite frequency and amplitude.

For general application, Proportional Derivatives (PD) and Proportional Integrator Derivatives (PID) control techniques continue to be widely used due to their simplicity, real-time implementation and no mathematical model requirement (e.g., He and Zhao, 2014; Nguyen and Hong, 2018). Recently, has proposed a PID approach control for enhancement of a DJI-F450 drone model. They are also associated with other control techniques to improve their efficiency and reduce their disadvantages. To compensate disturbances in attitude and altitude control, a PID and fuzzy – PID controls combination was proposed by Kuantama et al. (2017). Using predictive models and linear quadratic optimisation techniques, PID controllers in discrete version have also been investigated by Khan and Kadri (2015) and Reizenstein (2017). In fact, a PID controller cannot cover all fluctuations in the dynamics of a system. However, it can cover a significant amount of uncertainty and ensure fast control response. This is crucial for aerial quadrotors controlling attitude to avoid obstacles and perform demanding maneuvers in urban or forest areas. PID control has been combined with a variety of tuning approaches in several studies in order to improve its efficiency. Auto-tuning algorithms using metaheuristics deep reinforcement learning, neural network or fuzzy rules are recently explored (e.g., Doukhi et al., 2017; Sheta et al., 2021). Basically, the PD-PID controllers present the advantage of having only two effects (for the PD controller) and three effects (for the PID controller) adjusted with the corresponding parameters. The system behavior could be modified with the forementioned parameters. In the existing literature, many works have been conducted for the evaluation of these parameters without necessarily appealing a mathematical model of the system. The effectiveness of controllers has long been established in the industry. Their utilization in more advanced systems such as robots and drones has also been demonstrated. In fact, their flexibility and simplicity made the PD-PID controllers gain popularity among researchers and practitioners.

There is no doubt that forest fire detection and surveillance are becoming increasingly important issues all over the world. The degradation of the ecosystem due to climate change is more and more visible. Several monitoring and management strategies involve the use of cooperative UAVs and Unmanned Ground Vehicles (UGVs) (e.g., Ghamry and Zhang, 2015; Bhar et al., 2017). Wild expanses to be explored require UAVs that are economical, have the capability of generating a trajectory, tracking, and avoiding obstacles (e.g., Ait El Cadi, 2010; Bhar et al., 2018).

The main contribution of this study is using simple PD with adjustable parameter controller associated with PID controller to make the quadrotor more stable when dealing with changes in weight and compensating for wind gusts. Our work involves designing a quadrotor UAV autopilot that will be used later in collaboration with intelligent navigation algorithms for surveillance and detection. By combining PD and PID controllers, the quadrotor autopilot offers a configurable architecture and a significant advantage over other autopilots. Through simulations under multiple disturbances, its performance were assessed.

The remainder of this article is organized as follows. Section 2 reports the motion mechanism and the dynamic model. Section 3 describes the design of the controller architecture. Section 4 details the Simulink parameters and reports the main numerical results. Conclusions and avenues for future work are drawn in Section 5.

## 2 Dynamic model

### 2.1 Quadrotor configuration

**Figure 1:**
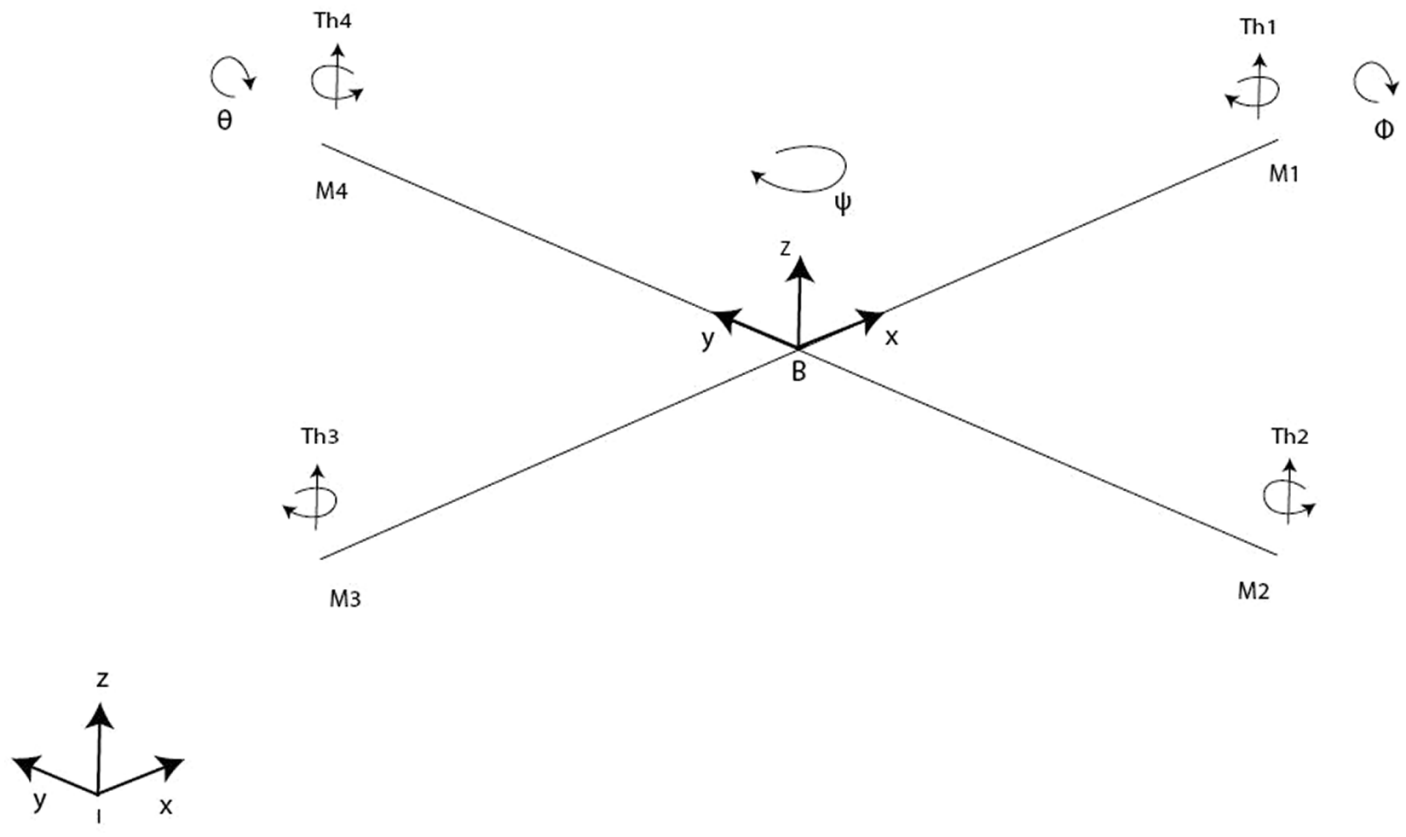
Quadrotor model.

A Quadrotor includes 4 rotors arranged in a cross-like shape as displayed in Figure 1. Rotor blades are equipped with propellers powered by one-by-one DC motors. Rotors 1 and 3 rotate within side the equal route at the same time as rotors 2 and 4 rotate in a contrary route in order to balance the whole machine torque and cancel the gyroscopic and aerodynamics torques.

### 2.2 Flight mechanisms

**Figure 2:**
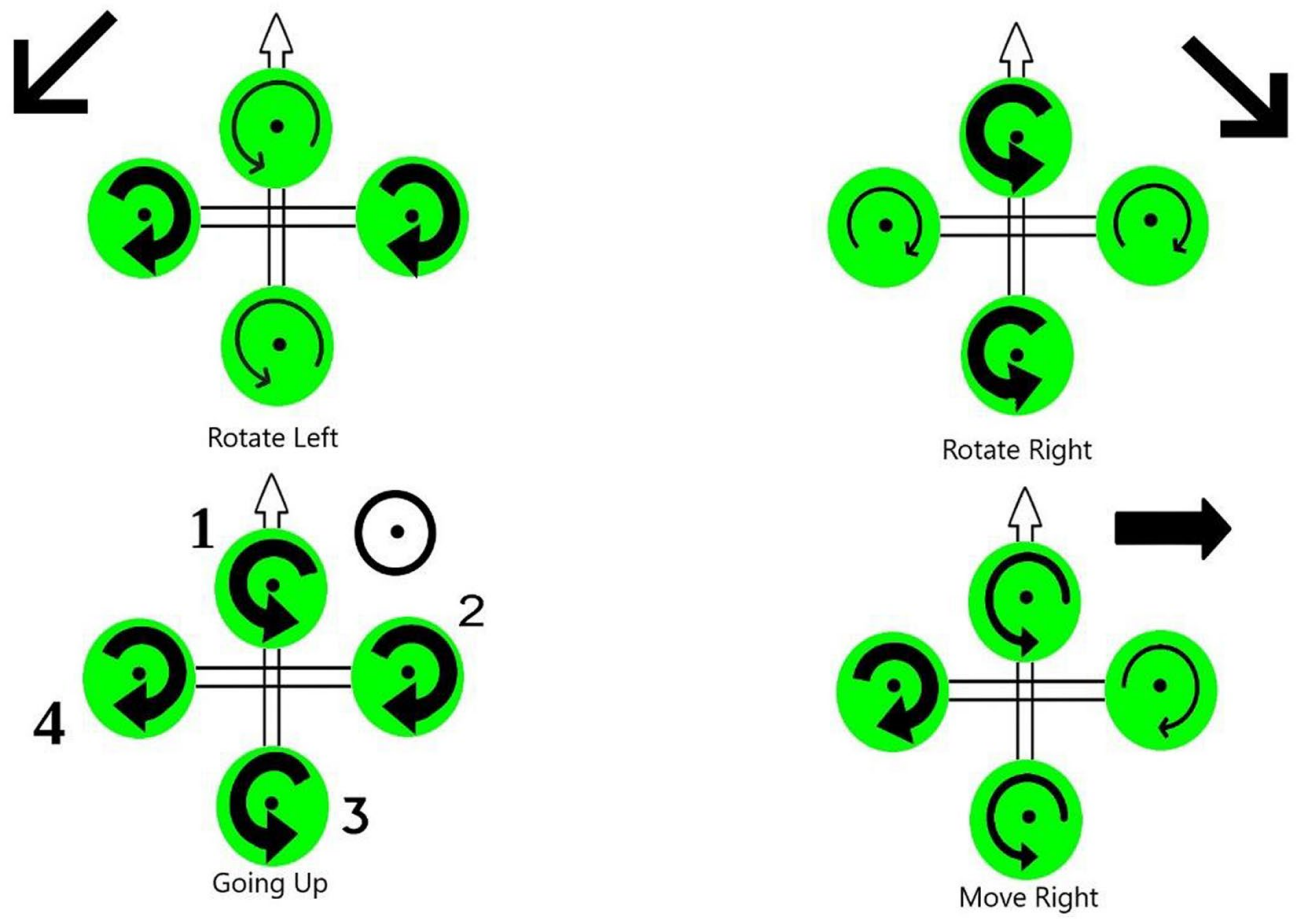
Quadrotor flight mechanisms.

The motion of quadcopters can be achieved through six distinct maneuvers, which involve a combination of translational and rotational motion as illustrated in Figure 2. There are four inputs on the quadcopter:

- Thrust (z): This input generates a vertical force that enables the quadcopter to hover or move up and down through the air. Increased or decreased speed of all four rotors by a similar amount, greater or lesser than gravitational force, creates the force.
- Roll angle (ϕ): It denotes the quadcopter rotation around the x-axis. In order to create the roll motion, the second rotor’s speed is decreased and the fourth rotor’s speed is increased. These “rolls” move the quadcopter sideways along the y + and y- axis, while maintaining its altitude position.
- Pitch (θ): It corresponds to the quadcopter rotation around the y-axis. By decreasing the first rotor’s speed and increasing the third rotor’s speed, pitch motion can be created. Depending on the quadcopter’s orientation and the position of its nose, the pitch action causes it to tilt upwards or downwards. An upward tilt moves the quadcopter in a backward motion (x-), while a downward tilt moves it forward (x+).
- Yaw (ψ): It refers to the quadcopter rotation around the z-axis. Angular velocities are increased of two opposite rotors while angular velocities of the other two are decreased. Thus, the Yaw motion is generated. Precisely, In both clockwise and counterclockwise rotations, the quadcopter’s front faces the same direction.

### 2.3 System coordinates

First, let us begin by precising the reference coordinate frame and the coordinate frame of the vehicle body, in order to construct the quadrotor’s mathematical model. Let us denote by B = [*B*x, *B*y, *B*z]^T^ the body-fixed frame (BFF) and (EFF) E = [E_x_, E_y_, E_z_]^T^ the ground fixed reference coordinate frame also called inertial frame. The center of the BFF origin is aligned with the center of mass of the quadcopter, and its origin is connected to the body of the vehicle.

In the 3-dimensional Euclidean space, we denote B = (x,y,z)^T^. The coordinate vector $\vec{IB}$ describes the distance between the Inertia frame and the body frame. The three Euler angles 𝜙, 𝜃 and 𝜓 (so called: roll, pitch, and yaw) describing the quadrotor orientation are represented by the vector Γ = [𝜙,,𝜓]^𝑇^.

The vehicle body frame BFF is considered the base for reference frame analysis. To catch the 3D space’s movements and create our mathematical references, Equations 1–5 are considered:

- Body frame rotated in 𝜓^+^:

(1) $$R_{B}^{I}\left(z,\psi \right)=\left[\begin{array}{ccc} \cos\psi & -\sin\psi & 0\\ \sin\psi & \cos\psi & 0\\ 0 & 0 & 1 \end{array}\right]$$

- Body frame rotated in 𝜙 +:

(2) $$R_{B}^{I}\left(x,\phi \right)=\left[\begin{array}{ccc} 1 & 0 & 0\\ 0 & \cos\phi & -\sin\phi \\ 0 & \sin\phi & \cos\phi \end{array}\right]$$

- Body frame rotated in 𝜃 +:

(3) $$R_{B}^{I}\left(y,\theta \right)=\left[\begin{array}{ccc} \cos\theta & 0 & \sin\theta \\ 0 & 1 & 0\\ -\sin\theta & 0 & \cos\theta \end{array}\right]$$

The movement from one frame to another in this case is defined by Equation 4:

(4) $$R_{B}^{I}\left(\psi ,\theta ,\phi \right)=R_{B}^{I}\left(z,\psi \right)R_{B}^{I}\left(y,\theta \right)R_{B}^{I}\left(x,\phi \right)$$

The rotational matrix is then derived as

(5) $$R_{B}^{I}=\left[\begin{array}{ccc} \cos\psi \cos\theta & \begin{array}{l} \sin\theta \sin\phi \cos\psi \\ -\cos\theta \sin\psi \end{array} & \begin{array}{l} \sin\theta \cos\phi \cos\psi \\ +\sin\phi \sin\psi \end{array}\\ \sin\psi \cos\theta & \begin{array}{l} \sin\theta \sin\phi \sin\psi \\ +\cos\phi \cos\psi \end{array} & \begin{array}{l} \sin\theta \cos\phi \sin\psi \\ -\sin\phi \cos\psi \end{array}\\ -\sin\theta & \sin\phi \cos\theta & \cos\phi \cos\theta \end{array}\right]$$

### 2.4 Actuator dynamics

The actuator dynamic refers to the correlation between the voltage applied to the actuator, which serves as the real control input, and the speed of the rotor. Designers are particularly interested in the speed of the actuator’s response, as it plays a crucial role in the performance of the system. Based on Kirchhoff laws and the law of rotation, we derive the following simplified actuator dynamic model described by Equation 6:

(6) $$\frac{dw_{i}}{dt}=-\frac{1}{\tau }w_{i}+\frac{K_{w}}{\tau }u_{i},\forall i=1,..,4$$

where 𝜏 and $K_{w}$ denotes the delay and gain coefficient, respectively (Bouabdallah et al., 2004). Furthermore, $\frac{dw_{i}}{dt}$ and $u_{i}$ represent the speed and the input voltage of actuator 𝑖, =1,..,4 . Actuator delay assumes a critical role, especially in cases where the attitude control loop is functioning at a low frequency.

### 2.5 Kinematics

Let us move on to the kinematics side of the conducted study. Herein, the rigid “+” symmetric quadrotor is shown in Figure 1 with four rotors producing (Th1, Th2, Th3, Th4) forces. Quadrotor structure center of mass and origin of body coordinate system are the same. The center of gravity of the system coincides with the origin of the body-fixed frame (BFF). It is assumed that propellers are rigid and that thrust and drag are proportional to the square of the propeller’s rotational speed. The difference between the body rates p, q, r in BFF and the angle rates expressed in EFF should be calculated following Equations 7, 8.

(7) $$\left[\begin{array}{c} p\\ q\\ r \end{array}\right]=\left[\begin{array}{ccc} 1 & 0 & -\sin\theta \\ 0 & \cos\theta & \sin\phi \cos\theta \\ 0 & -\sin\theta & \cos\phi \cos\theta \end{array}\right]\left[\begin{array}{c} \dot{\phi }\\ \dot{\theta }\\ \dot{\psi } \end{array}\right]$$

(8) $$v=M\dot{\Gamma }$$

Where v and $\dot{\Gamma }$ represent the angular velocity vectors in both frames the B-frame and E-frame, respectively. Furthermore, the inverse of the matrix M is expressed in Equation 9 as

(9) $$M^{-1}=\left[\begin{array}{ccc} 1 & \tan\theta \sin\phi & \tan\theta \cos\phi \\ 0 & \cos\phi & -\sin\phi \\ 0 & \frac{\sin\phi }{\cos\theta } & \frac{\cos\phi }{\cos\theta } \end{array}\right]$$

During position control, the quadrotor’s body must be compensated for rotation. Compensation is obtained by transposing the rotation matrix.

#### 2.5.1 Translation dynamics

Based on the works of Barzegar and Lee (2022) and Quan (2017), the system under study was subjected to the following forces:

The vehicle’s total weight:

(10) $$P=\left[\begin{array}{c} 0\\ 0\\ -\mathrm{mg} \end{array}\right]$$

The rotors’ generated thrust:

(11) $$F_{th}=R_{B}^{I}\overset{4}{\underset{i=1}{\sum }}Th_{i}=b\overset{4}{\underset{i=1}{\sum }}{\omega_{i}}^{2}\left[\begin{array}{c} \sin\phi \sin\psi +\sin\theta \cos\psi \cos\phi \\ -\sin\phi \cos\psi +\sin\psi \cos\phi \sin\theta \\ \cos\theta \cos\phi \end{array}\right]$$

The drag force and air friction:

(12) $$F_{dr}=C_{d}\dot{B}=-\left[\begin{array}{ccc} C_{dx} & 0 & 0\\ 0 & C_{\mathrm{dy}} & 0\\ 0 & 0 & C_{dz} \end{array}\right]\left[\begin{array}{c} \dot{x}\\ \dot{y}\\ \dot{z} \end{array}\right]=\left[\begin{array}{c} C_{dx}\dot{x}\\ C_{\mathrm{dy}}\dot{y}\\ C_{dz}\dot{z} \end{array}\right]$$

(13) $$F=m\ddot{B}=P+F_{th}+F_{dr}$$

The mass of the quadrotor is represented by m and the acceleration due to gravity is represented by g in Equation 10. Equation 11 expresses that the angular velocity and thrust constant of the i^th^ propeller are represented by $\omega_{i}$ and b , respectively. Equation 12 contains the translational drag coefficient matrix denoted by $C_{d}$ . The position of the center of mass in the coordinates of the Flat Earth, as given in Equation 10, is a vector of the dimensions 3 times 1. Then, the total force is expressed by Equation 13. The equation of motion describing the translational motion of the quadcopter is expressed using Newton’s second law and reads are Equations 14–16:

(14) $$\ddot{x}=\frac{1}{m}\left(b\overset{4}{\underset{i=1}{\sum }}{\omega_{i}}^{2}\left(\sin\phi \sin\psi +\cos\phi \sin\theta \cos\psi \right)-C_{dx}\dot{x}\right)$$

(15) $$\ddot{y}=\frac{1}{m}\left(b\overset{4}{\underset{i=1}{\sum }}{\omega_{i}}^{2}\left(-\sin\phi \cos\psi +\sin\psi \cos\phi \sin\theta \right)-C_{\mathrm{dy}}\dot{y}\right)$$

(16) $$\ddot{z}=\frac{1}{m}\left(b\overset{4}{\underset{i=1}{\sum }}{\omega_{i}}^{2}\left(\cos\theta \cos\phi \right)-C_{dz}\dot{z}-\mathrm{mg}\right)$$

#### 2.5.2 Rotation dynamics

The motion of a quadrotor is clearly attributed to an array of forces and moments resulting from various physical phenomena. A quadrotor experiences torques in roll, pitch, and yaw, as well as aerodynamic friction torque and gyroscopic effects caused by the propellers. To calculate angular acceleration on the body frame, the second law of Newton for rotational motion must be applied, and the Coriolis effect should be taken into account. We can consider for this model the torques detailed in Table 1.

**Table 1:** Torques details.

Type	Designation	Expression
Rolling moments ( $\tau_{x})$	Body gyro effect	$\dot{\theta }\dot{\psi }\left(I_{y}-I_{z}\right)$
	Propeller gyro effect	$J_{r}\dot{\theta }\Omega_{r}$
	Roll actuators action	$l\left(-Th_{2}+Th_{4}\right)$
	Rolling moment due to air resistance	$C_{\mathrm{ax}}{\dot{\phi }}^{2}$
Pitching moments ( $\tau_{y})$	Body gyro effect	$\dot{\phi }\dot{\psi }\left(I_{z}-I_{x}\right)$
	Propeller gyro effect	$J_{r}\dot{\phi }\Omega_{r}$
	Pitch actuators action	$l\left(Th_{1}-Th_{3}\right)$
	Pitch moment due to air resistance	$C_{ay}{\dot{\theta }}^{2}$
Yawing moments ( $\tau_{z})$	Body gyro effect	$\dot{\theta }\dot{\phi }\left(I_{x}-I_{y}\right)$
	Inertial counter-torque	$J_{r\dot{\Omega }r}$
	Counter-torque unbalance	${\left(-1\right)}^{i}\overset{4}{\underset{i=1}{\sum }}Q_{i}$
	Yaw moment due to air resistance	$C_{az}{\dot{\psi }}^{2}$

Accordingly, the torques can be derived as follows:

(17) $$\tau_{x}=\left[\begin{array}{c} 0\\ -l\\ 0 \end{array}\right]\wedge \left[\begin{array}{c} 0\\ 0\\ Th_{2} \end{array}\right]+\left[\begin{array}{c} 0\\ l\\ 0 \end{array}\right]\wedge \left[\begin{array}{c} 0\\ 0\\ Th_{4} \end{array}\right]=\left[\begin{array}{c} \mathrm{lb}\left({\omega_{4}}^{2}-{\omega_{2}}^{2}\right)\\ 0\\ 0 \end{array}\right]$$

(18) $$\tau_{y}=\left[\begin{array}{c} l\\ 0\\ 0 \end{array}\right]\wedge \left[\begin{array}{c} 0\\ 0\\ Th_{1} \end{array}\right]+\left[\begin{array}{c} -l\\ 0\\ 0 \end{array}\right]\wedge \left[\begin{array}{c} 0\\ 0\\ Th_{3} \end{array}\right]=\left[\begin{array}{c} 0\\ \mathrm{lb}\left({\omega_{3}}^{2}-{\omega_{1}}^{2}\right)\\ 0 \end{array}\right]$$

(19) $$\tau_{z}=\left[\begin{array}{c} 0\\ 0\\ d\overset{4}{\underset{i=1}{\sum }}{\omega_{i}}^{2} \end{array}\right]$$

(20) $$\tau_{a}=C_{a}\left[\begin{array}{c} {\dot{\psi }}^{2}\\ {\dot{\phi }}^{2}\\ {\dot{\theta }}^{2} \end{array}\right]=\left[\begin{array}{c} C_{az}{\dot{\psi }}^{2}\\ C_{\mathrm{ax}}{\dot{\phi }}^{2}\\ C_{ay}{\dot{\theta }}^{2} \end{array}\right]$$

(21) $$\tau_{gp}=J_{r}\Omega_{r}\left[\begin{array}{c} 0\\ \dot{\theta }\\ -\dot{\phi } \end{array}\right]$$

Equations 17–21 denote that the parameter l indicates the distance of the motor axis from the center of gravity of the quadcopter, while d represents the drag coefficient. Equation 20 depicts $C_{a}$ as the 3×3 matrix of aerodynamic friction coefficients. In Equation 21, $J_{r}$ and $\Omega_{r}$ represent the rotor’s inertia and rotation velocity, respectively. The rotational motion of the quadrotor is governed by the equations of motion, expressed by Equations 22–24, obtained by using Euler’s rotation equations. In Equations 22–24, moments of inertia along x , y and z directions are represented by $I_{x}$ , $I_{y}$ and $I_{z}$ respectively:

(22) $$\ddot{\phi }=\frac{1}{I_{x}}\left(-C_{\mathrm{ax}}{\dot{\phi }}^{2}-J_{r}\Omega_{r}\dot{\theta }-\left(I_{z}-I_{y}\right)\dot{\theta }\dot{\psi }+\mathrm{lb}\left({\omega_{4}}^{2}-{\omega_{2}}^{2}\right)\right)$$

(23) $$\ddot{\theta }=\frac{1}{I_{y}}\left(-C_{ay}{\dot{\theta }}^{2}-J_{r}\Omega_{r}\dot{\phi }-\left(I_{x}-I_{z}\right)\dot{\phi }\dot{\psi }+\mathrm{lb}\left({\omega_{3}}^{2}-{\omega_{1}}^{2}\right)\right)$$

(24) $$\ddot{\psi }=\frac{1}{I_{z}}\left(-C_{ay}{\dot{\theta }}^{2}-J_{r}\Omega_{r}\dot{\phi }-\left(I_{x}-I_{z}\right)\dot{\phi }\dot{\psi }+\overset{4}{\underset{i=1}{\sum }}{\left(-1\right)}^{i+1}{\omega_{i}}^{2}\right)$$

#### 2.5.3 Mathematical model

Considering both the translational and rotational dynamics, the entire dynamic model of the quadcopter is expressed as follows:

(25) $$\ddot{x}=\frac{1}{m}\left(u_{z}u_{x}-C_{dx}\dot{x}\right)$$

(26) $$\ddot{y}=\frac{1}{m}\left(u_{z}u_{y}-C_{\mathrm{dy}}\dot{y}\right)$$

(27) $$\ddot{z}=\frac{1}{m}\left(u_{z}\left(\cos\theta \cos\phi \right)-C_{dz}\dot{z}-\mathrm{mg}\right)$$

(28) $$\ddot{\phi }=\frac{1}{I_{x}}\left(-C_{\mathrm{ax}}{\dot{\phi }}^{2}-J_{r}\Omega_{r}\dot{\theta }-\left(I_{z}-I_{y}\right)\dot{\theta }\dot{\psi }+u_{\phi }\right)$$

(29) $$\ddot{\theta }=\frac{1}{I_{y}}\left(-C_{ay}{\dot{\theta }}^{2}-J_{r}\Omega_{r}\dot{\phi }-\left(I_{x}-I_{z}\right)\dot{\phi }\dot{\psi }+u_{\theta }\right)$$

(30) $$\ddot{\psi }=\frac{1}{I_{z}}\left(-C_{ay}{\dot{\theta }}^{2}-J_{r}\Omega_{r}\dot{\phi }-\left(I_{x}-I_{z}\right)\dot{\phi }\dot{\psi }+u_{\psi }\right)$$

where $u=\left(u_{x},u_{y},u_{z},u_{\phi },u_{\theta },u_{\psi }\right)$ is a vector expressed by Equations 31–33 with d as the drag coefficient:

(31) $$u_{x}=\sin\phi \sin\psi +\cos\phi \sin\theta \cos\psi$$

(32) $$u_{y}=-\sin\phi \cos\psi +\sin\psi \cos\phi \sin\theta$$

(33) $$\left[\begin{array}{c} u_{z}\\ u_{\phi }\\ \begin{array}{c} u_{\theta }\\ u_{\psi } \end{array} \end{array}\right]=\left[\begin{array}{c} b\;b\;\begin{array}{cc} b & b \end{array}\\ \begin{array}{ccc} 0 & -\mathrm{lb} & \begin{array}{cc} 0 & \mathrm{lb} \end{array} \end{array}\\ \begin{array}{c} \begin{array}{ccc} -\mathrm{lb} & 0 & \begin{array}{cc} \mathrm{lb} & 0 \end{array} \end{array}\\ \begin{array}{ccc} d & -d & \begin{array}{cc} d & -d \end{array} \end{array} \end{array} \end{array}\right]\left[\begin{array}{c} {\omega_{1}}^{2}\\ {\omega_{2}}^{2}\\ \begin{array}{c} {\omega_{3}}^{2}\\ {\omega_{4}}^{2} \end{array} \end{array}\right]$$

Equations 25–30 exhibit the complete nonlinear model of a quadrotor, revealing its complex dynamics. The strong nonlinearity, including inter-state multiplication, intensive variable coupling, and the presence of multivariable features, poses challenges in designing controllers, while simultaneously eliciting significant researchers’ attention.

#### 2.5.4 Simplified system

The purpose of the mathematical model is to facilitate the construction of a control system. Nevertheless, if the mathematical model is overly sophisticated, the design of the control system will also be complex. A state space model could be adapted for the control system $\dot{X}=f\left(X,U\right)$ where X and U are the state and input vectors, respectively. The state vector X reads as:

(34) $$X=\left(x,y,z,\dot{x},\dot{y},\dot{z,}\phi ,\theta ,\psi ,\dot{\phi },\dot{\theta },\dot{\psi }\right)$$

Let us begin by assuming that

(35) $$\left\{ \;\begin{array}{c} \begin{array}{c} x_{1}=x\\ x_{2}=y\\ x_{3}=z \end{array}\\ \begin{array}{c} \begin{array}{c} x_{4}=\dot{x}={\dot{x}}_{1}\\ x_{5}=\dot{y}={\dot{x}}_{2}\\ x_{6}=\dot{z}={\dot{x}}_{3} \end{array}\\ \begin{array}{c} \;x_{7}=\phi \\ x_{8}=\theta \\ x_{9}=\psi \end{array}\\ x_{10}=\dot{\phi }={\dot{x}}_{7} \end{array}\\ \begin{array}{c} x_{11}=\dot{\theta }={\dot{x}}_{8}\\ x_{12}=\dot{\psi }={\dot{x}}_{9} \end{array} \end{array}\right.$$

Then, Equations 25–40 leads to

(36) $$\dot{X}=\left[\;\begin{array}{l} x_{4}\\ \;x_{5}\\ \;x_{6}\\ \;\frac{1}{m}\left(u_{z}u_{x-C_{dx}\dot{x}}\right)\\ \;\frac{1}{m}\left(u_{z}u_{y-C_{\mathrm{dy}}\dot{y}}\right)\\ \;\frac{1}{m}\left(u_{z}\left(\cos\theta \cos\phi \right)-C_{dz}\dot{z}-\mathrm{mg}\right)\\ \;x_{10}\\ \;x_{11}\\ \;x_{12}\\ \frac{1}{1_{x}}\left(-C_{\mathrm{ax}}{\dot{\phi }}^{2}-J_{r}\Omega_{r}\dot{\theta }-\left(I_{z}-I_{y}\right)\dot{\theta }\dot{\psi }+u_{\phi }\right)\\ \frac{1}{1_{y}}\left(-C_{ay}{\dot{\theta }}^{2}-J_{r}\Omega_{r}\dot{\phi }-\left(I_{x}-I_{z}\right)\dot{\phi }\dot{\psi }+u_{\theta }\right)\\ \frac{1}{1_{z}}\left(-C_{ay}{\dot{\theta }}^{2}-J_{r}\Omega_{r}\dot{\phi }-\left(I_{x}-I_{z}\right)\dot{\phi }\dot{\psi }+u_{\psi }\right) \end{array}\right]$$

Equation 36 indicates that rotational movements are functionally independent of translational movements and are entirely actuated. However, translational movements are under actuated and highly dependent on rotational movements. A control loop structure comprising both an inner and outer loop has been devised. The inner control loop guarantees asymptotic tracking of the desired attitude and altitude, ensuring precise control. On the other hand, the outer loop is responsible for conducting navigation, overseeing the path planning and overall trajectory of the system.

It is possible to make certain simplifying assumptions for the previous model, without compromising the precision of the motion behavior to a significant extent. To minimize the complexity of calculations, it is presumed that the quadrotor is near a hovering position, with very little changes in angles and angular acceleration contribution. Resulting in a null attitude angular rates ( $\dot{\phi }=0$ , $\dot{\theta }=0$ , $\dot{\psi }=0$ ), this enables neglecting of the gyroscopic effects and moments of inertia terms. Additionally, assuming that the angles ϕ and θ are small and the Coriolis terms can be neglected, we can consider that the Coriolis effect terms do not exert a significant influence on linear acceleration. Additionally, considering the quadcopter’s movement near a hovering state allows the approximation of the velocities transformation matrix M between the BFF and EFF frames to be close to identity.

Thereby, the motion Equation 36 becomes

(37) $$\dot{X}=\left[\;\begin{array}{c} \begin{array}{c} x_{4}\\ x_{5}\\ x_{6} \end{array}\\ \begin{array}{c} \begin{array}{c} \frac{1}{m}\left(u_{z}u_{x}\right)\\ \frac{1}{m}\left(u_{z}u_{y}\right)\\ \frac{1}{m}\left(u_{z}\left(\cos\theta \cos\phi \right)-\mathrm{mg}\right) \end{array}\\ \begin{array}{c} \;x_{10}\\ x_{11}\\ x_{12} \end{array}\\ \frac{u_{\phi }}{I_{x}} \end{array}\\ \begin{array}{c} \frac{u_{\theta }}{I_{y}}\\ \frac{u_{\psi }}{I_{z}} \end{array} \end{array}\right]\text{.}$$

## 3 Controller design

In this section, the control strategy and design of the attitude and elevation controller are described in detail.

### 3.1 Control strategy

**Figure 3:**
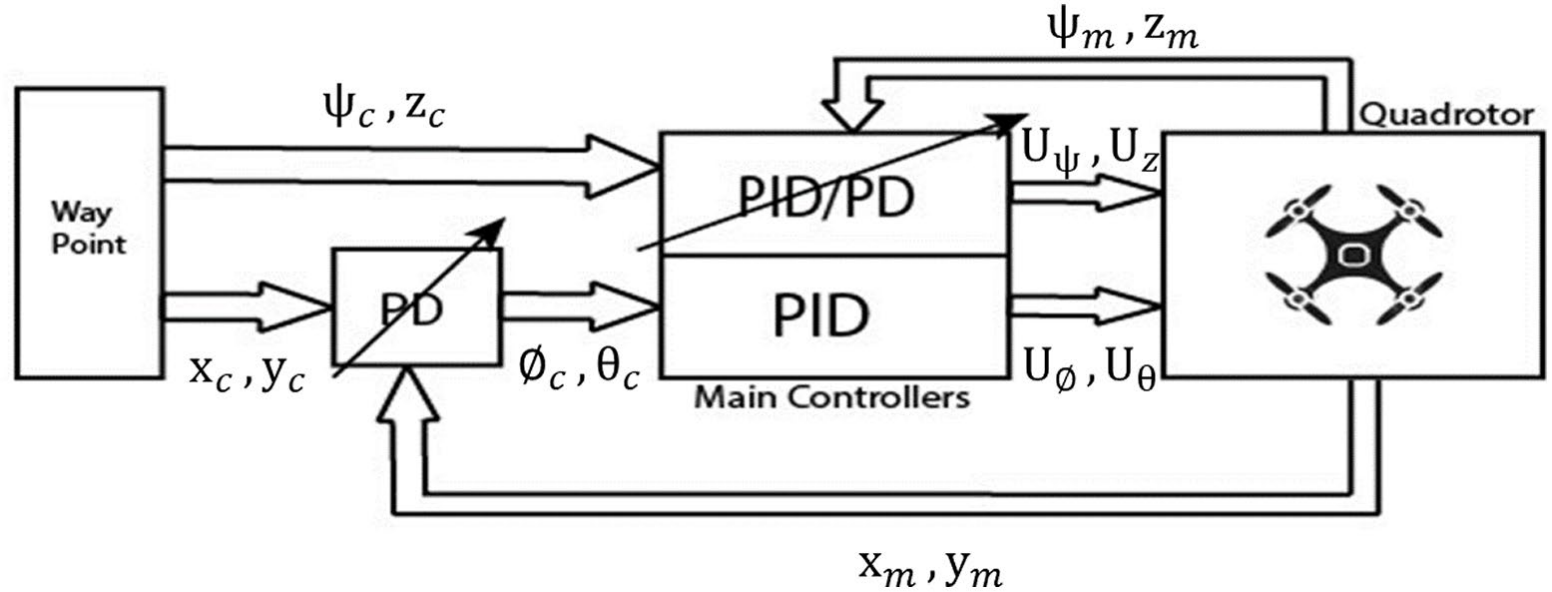
The full quadrotor control scheme.

Achieving seamless tracking performance utilizing traditional gains is the primary goal of the proposed PD-PID controller, while simultaneously ensuring satisfactory performance in position hold mode through gain adjustment aimed at mitigating the effects of wind disturbances. A schematic representation of the proposed PD-PID technique is depicted in Figure 3. The proportional, integral, and derivative gains are optimized utilizing gain scheduling, which is determined by variations in natural frequency and the Ziegler-Nichols methods.

### 3.2 Attitude control

The primary control loop, as proposed, governs four Degrees of Freedom (DOF) in the quadrotor system, namely the altitude z , roll angle ϕ , pitch angle θ , and yaw angle ψ . Given that the quadrotor dynamic system is under-actuated, an outer loop is employed to derive the desired control inputs for roll and pitch, denoted as $\phi_{d}$ and $\theta_{d}$ , respectively, based on the desired positions in the earth frame $\left(x_{e},y_{e}\right)$ . For the linear acceleration, very small angles ϕ and θ are assumed. Inspired from the work of Kotarski et al. (2016), the following desired angles are derived:

(38) $$\left[\begin{array}{c} \phi_{d}\\ \theta_{d} \end{array}\right]=\frac{1}{g}\left[\begin{array}{cc} \sin\psi & -\cos\psi \\ \cos\psi & \sin\psi \end{array}\right]\left[\begin{array}{c} T_{x}\\ T_{y} \end{array}\right]$$

A configurable PD controller is also envisaged, following the work of Nguyen and Hong (2018). the controller produces virtual accelerations $T_{x}$ and $T_{y}$ along the X and Y axes, respectively, such as

(39) $$T_{x}=k_{1x}\left(k_{2x}\left(x_{d}-x\right)-\dot{x}\right)$$

(40) $$T_{y}=k_{1y}\left(k_{2y}\left(y_{d}-y\right)-\dot{y}\right)$$

where the coordinates ( $x_{d},y_{d})$ refer to the desired position in the $\left(X,Y\right)$ plane, whereas ( $k_{1x}$ , $k_{2x})$ and ( $k_{1y}$ , $k_{2y}$ ) represent the controller gains along the X and Y axes, respectively.

**Figure 4:**
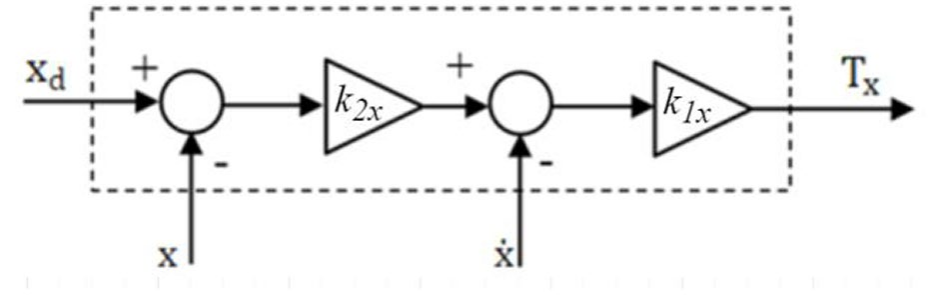
X-direction PD roll controller.

To summarize, the block diagram shown in Figure 4 represents the roll controller for the X-axis. It is evident that the structure of the roll controller for the Y direction is highly similar.

Now, let ξ and $w_{n}$ denote and the controller’s damping ratio and the natural frequency, respectively. Accordingly, Equation 41 was derived as a transfer function of second order that leads to the controller gains $k_{1x}$ and $k_{2x}$ , expressed by Equations 42, 43, respectively:

(41) $$\frac{T_{x}\left(p\right)}{X_{d}\left(p\right)}=\frac{k_{1x}k_{2x}}{p^{2}+k_{1x}p+k_{1x}k_{2x}}$$

(42) $$k_{1x}=2\xi w_{n}$$

(43) $$k_{2x}=\frac{w_{n}}{2\xi }$$

### 3.3 Roll control

To facilitate roll control and achieve motion along the Y-axis, the PID controller is utilized to generate small variations in forces which are then applied to rotors 3 and 4. If we assume that the roll parameters of the PID controller are represented by $K_{P\phi }$ , $K_{I\phi }$ , and $K_{D\phi }$ , and that the actual roll angle is $\phi_{m}$ , then the roll angle error $e_{\phi }$ can be expressed as $\phi_{d}-\phi_{m}$ . Accordingly, $\Delta Th_{\phi }$ can be represented by Equation 44:

(44) $$\Delta Th_{\phi }=K_{P\phi }e_{\phi }+K_{I\phi }\int e_{\phi }+K_{D\phi }{\dot{e}}_{\phi }$$

### 3.4 Pitch control

In order to generate motion along the X-axis, a PID controller is employed to generate a small variation in forces, denoted as $\Delta Th_{\theta }$ , which is then applied to rotors 1 and 2. If the quadrotor needs to move backwards, the speed of rotor 2 is decreased while the speed of rotor 1 is increased. This differential change in rotational speeds causes the quadrotor to move in a backward direction along the X-axis. This speed variation is controlled by PID which pitch angle parameters are $K_{P\theta }$ , $K_{I\theta }$ , and $K_{D\theta }$ so that $\Delta Th_{\phi }$ is expressed according to Equation 45:

(45) $$\Delta Th_{\theta }=K_{P\theta }e_{\theta }+K_{I\theta }\int e+K_{D\theta }{\dot{e}}_{\theta }$$

and $e_{\theta }=\theta_{d}-\theta_{m}$ , such that the pitch angle error $e_{\theta }$ is associated with the measured pitch angle $\theta_{m}$ .

### 3.5 Yaw control

The quadrotor’s counter clockwise and clockwise movements can be controlled by altering the yaw angle. The yaw angle parameters of the considered PD or PID controller are $K_{P\psi }$ , $K_{I\psi }$ , and $K_{D\psi }$ . Additionally, $\psi_{m}$ represents the sensor measured yaw angle, and $e_{\psi }$ represents the yaw angle error. Thus, as with the roll and pitch control, $e_{\psi }$ equals $\psi_{d}-\psi_{m}$ . Equations 46, 47 represent the yaw control equations for the PID and PD controllers, respectively:

(46) $$\Delta Th_{\psi }=K_{P\psi }e_{\psi }+K_{I\psi }\int e_{\psi }+K_{D\psi }{\dot{e}}_{\psi }$$

(47) $$\Delta Th\psi =K_{P\psi }e_{\psi }+K_{D\psi }{\dot{e}}_{\psi }$$

### 3.6 Altitude control

A thrust force is created to compensate for the gravitational force. Altering the angular velocities of all rotors at equal speed is the way to achieve the desired altitude of the quadrotor. Let $K_{Pz}$ , $K_{Iz}$ , and $K_{Dz}$ denote the altitude parameters of the PID controller, $z_{m}$ be the sensor measured altitude, and $e_{z}$ be the altitude error. Obviously, $e_{z}$ equals $z_{d}-z_{m}$ . According to these notations, the thrust forces for the PID and PD controllers, respectively, could be expressed by Equations 48, 49 as

(48) $$\Delta Th_{z}=K_{Pz}e_{z}+K_{Iz}\int e_{z}+K_{Dz}{\dot{e}}_{z}$$

(49) $$\Delta Th_{z}=K_{Pz}e_{z}+K_{Dz}{\dot{e}}_{z}$$

## 4 Simulation and experiments

Using Matlab Simulink, we implemented the simulation study, the experiments, and the parameter tuning for the PD and PID controllers (Figure 3). The control scheme can be generally characterized as being comprised of a waypoint input, which in turn is divided into a position component and a yaw angle orientation component. In the upcoming subsections, we will provide concise descriptions of the primary controllers and quadrotor parameters. Subsequently, we will present simulation results pertaining to main movements and trajectory tracking. Finally, we will evaluate control robustness by examining the quadrotor’s response to wind disturbances across various trajectories.

### 4.1 Controllers and quadrotor parameters setting

For the simulation study, we have adopted a quadrotor which main parameters are displayed in Table 2.

**Table 2:** Main quadrotor parameters.

Parameter	Value	Unit
L	0.24	m
m	0.9	Kg
g	9.81	m.s^−2^
d	3.59 10^−5^	N.m
$J_{x}$	0.00963	Kg.m^2^
$J_{y}$	0.00963	Kg.m^2^
$J_{z}$	0.019	Kg.m^2^

For the design of the quadrotor autopilot, a PD/PID controller is implemented as presented in Section 3. As a result of Ziegler-Nichols PID parameters setting rules, the proportion and derivative coefficients of the controller are adjusted. According to this parameter-setting method, parameters are set according to stability analysis. Therefore, $K_{p}$ the proportion coefficient is set such that $K_{D}$ and $K_{I}$ are fixed to 0. Then, an iterative increase in $K_{p}$ is made until the closed-loop system begins to shock, i.e., as soon as the poles of the closed-loop system cross the 𝑗𝑤 axis. Equations 50–52 express the final $K_{p}$ , $K_{D}$ , and $K_{I}$ values as

(50) $$K_{p}=0.6K_{m}$$

(51) $$K_{D}=\frac{{K_{P}}_{\pi }}{4w_{m}}$$

(52) $$K_{I}=\frac{K_{P}w_{m}}{\pi }$$

where the value of $K_{p}$ at which the system experiences shock is represented by $K_{m}$ , while $w_{m}$ denotes the frequency of oscillation. The root locus method is applied to determine $K_{m}$ and $w_{m}$ . The root locus of the controlled object’s transfer function can be obtained for a given system. $K_{m}$ corresponds to the gain traversing the 𝑗𝑤 axis, with the oscillation frequency of the point corresponding to $w_{m}$ .

### 4.2 Main movements

In order to assess and validate the PD and PID controller simulator, several numerical experiments were conducted.

**Figure 5:**
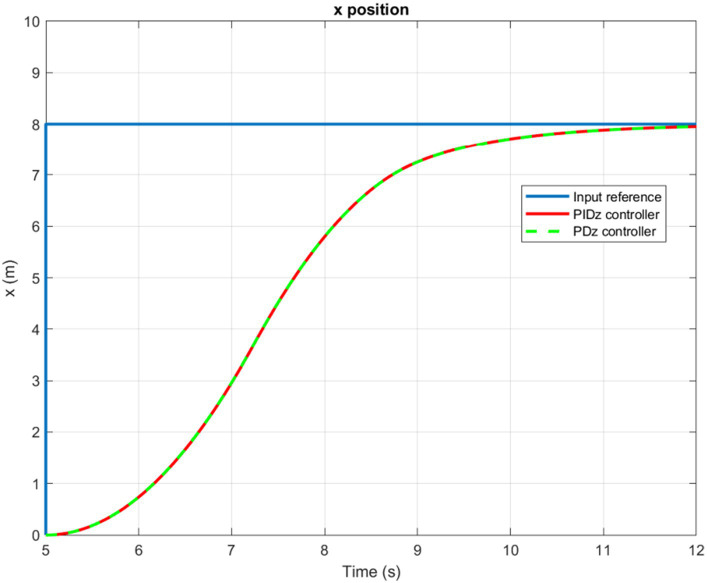
Time history of the X-position.

**Figure 6:**
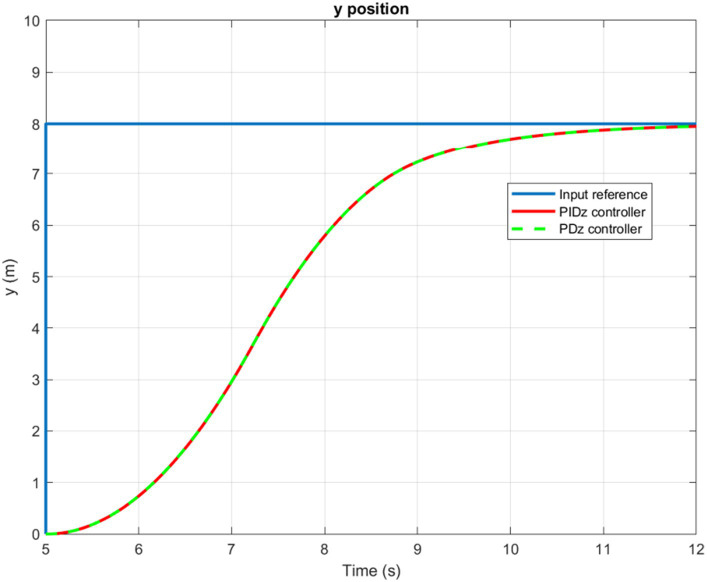
Time history of the Y-position.

**Figure 7:**
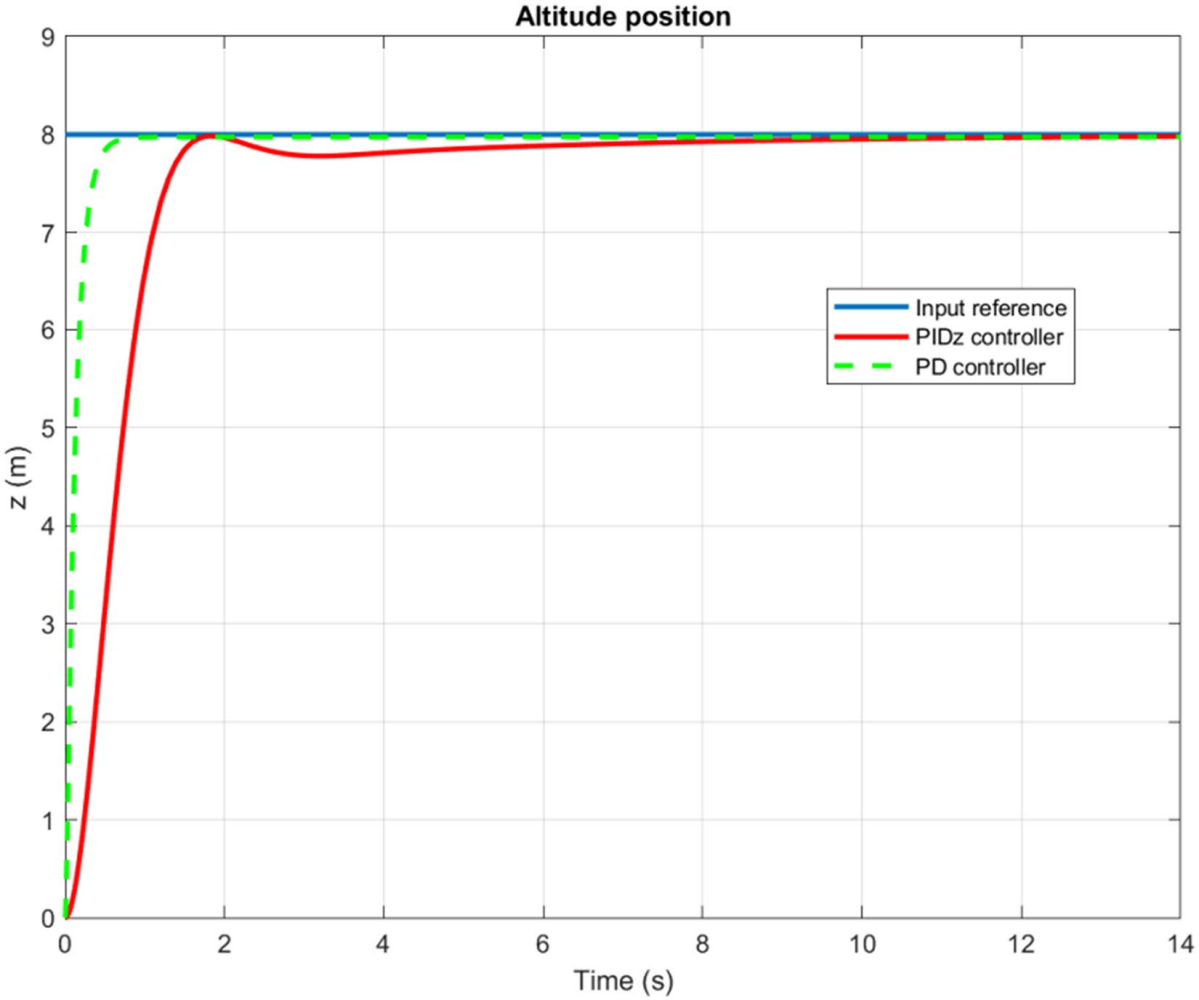
Time history of the Z-position.

**Figure 8:**
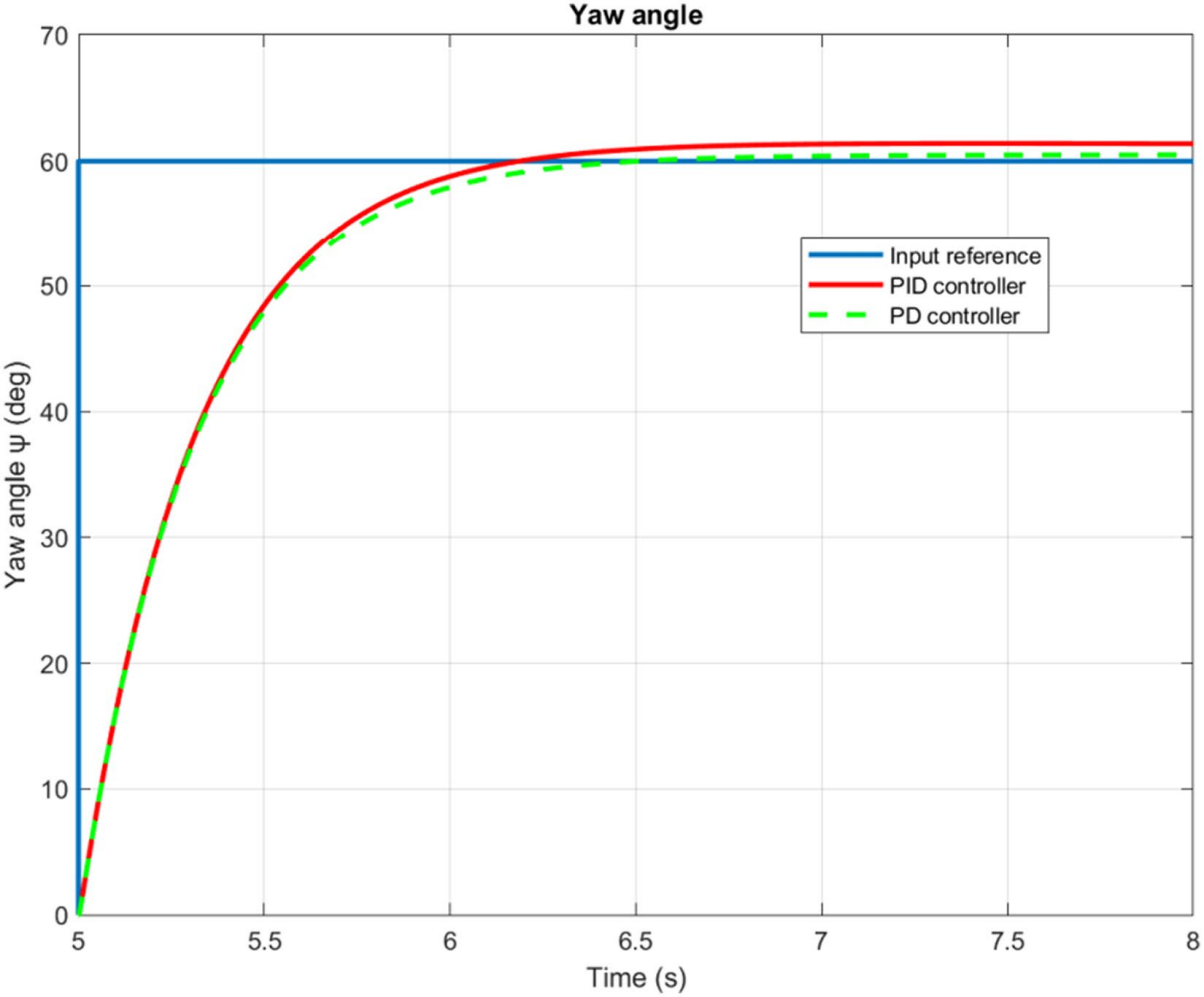
Quadrotor’s yaw angle.

The time evolution of the X, Y, Z, and Yaw angle positions of the tuned PD/PID controllers are reported in Figures 5–8. The PD and PID controllers have similar performance in terms of X and Y-axis motion, as shown in Figures 5, 6. It should also be noted that the Z position controller does not affect movement along the X and Y axes. The PID controller proves to be more efficient in reducing altitude position errors (Figure 7), whereas the PD controller only manages to keep a minor, permanent error. Additionally, the PD controller exhibits a marginally quicker rise time compared to its counterpart, the PID controller. The dynamics of the response of both PD/PID yaw controllers to the input reference show comparative behavior, as displayed in Figure 8.

**Figure 9:**
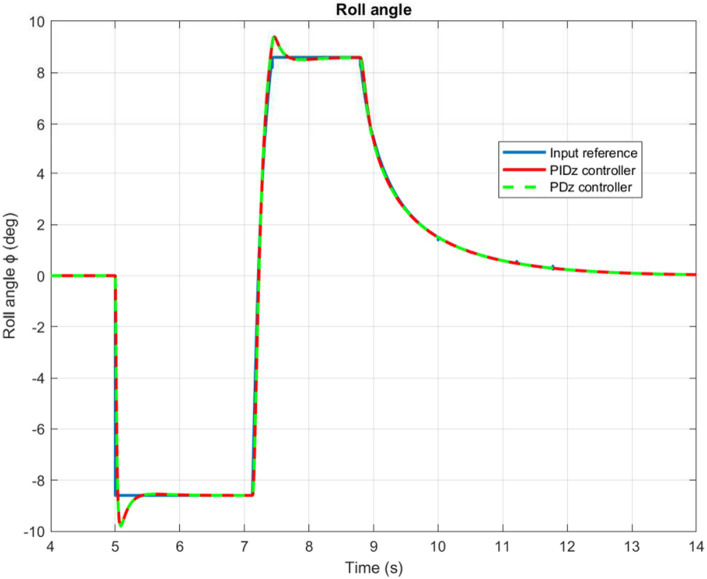
Quadrotor’s roll angle.

**Figure 10:**
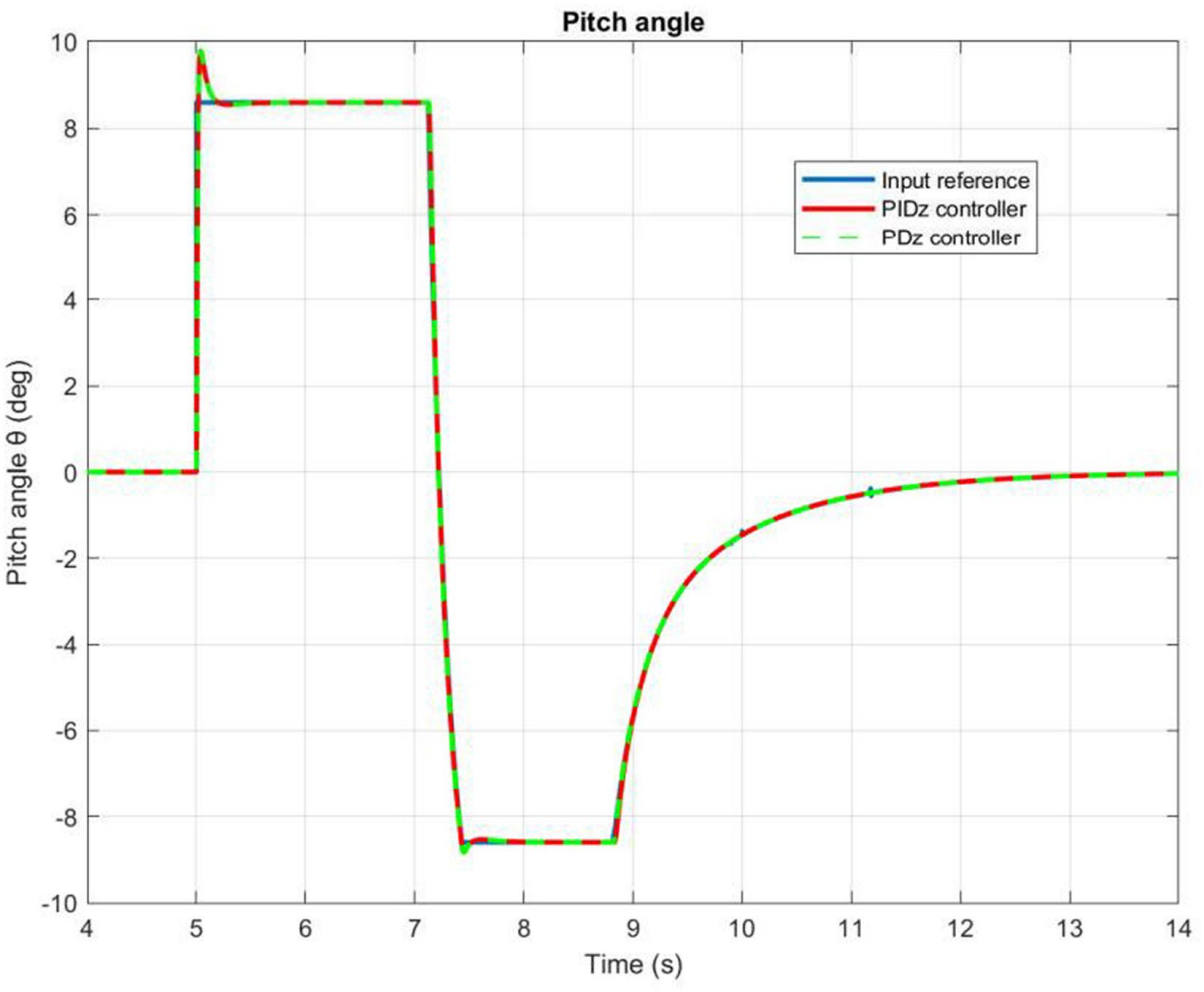
Quadrotor’s pitch angle.

Based on the information presented in Figures 9, 10, we can infer that the PID controlled inner loop has a quick response time as well as high degree of accuracy. The overshoot is well-damped, and the system stabilizes the roll and pitch input angles of the quadrotor. It succeeds to maintain these angles as minimal as possible, assuming negligible values for the ϕ and θ angles. In general, higher roll and pitch angles correlate with greater speeds along the X and Y directions.

### 4.3 Main results of the trajectory trucking

A wide range of signal shapes, such as square, circular, and sinusoidal trajectories, have been tested for trajectory trucking. In order to evaluate the performance of the proposed control architecture, a number of time-varying input references were examined and implemented.

**Figure 11:**
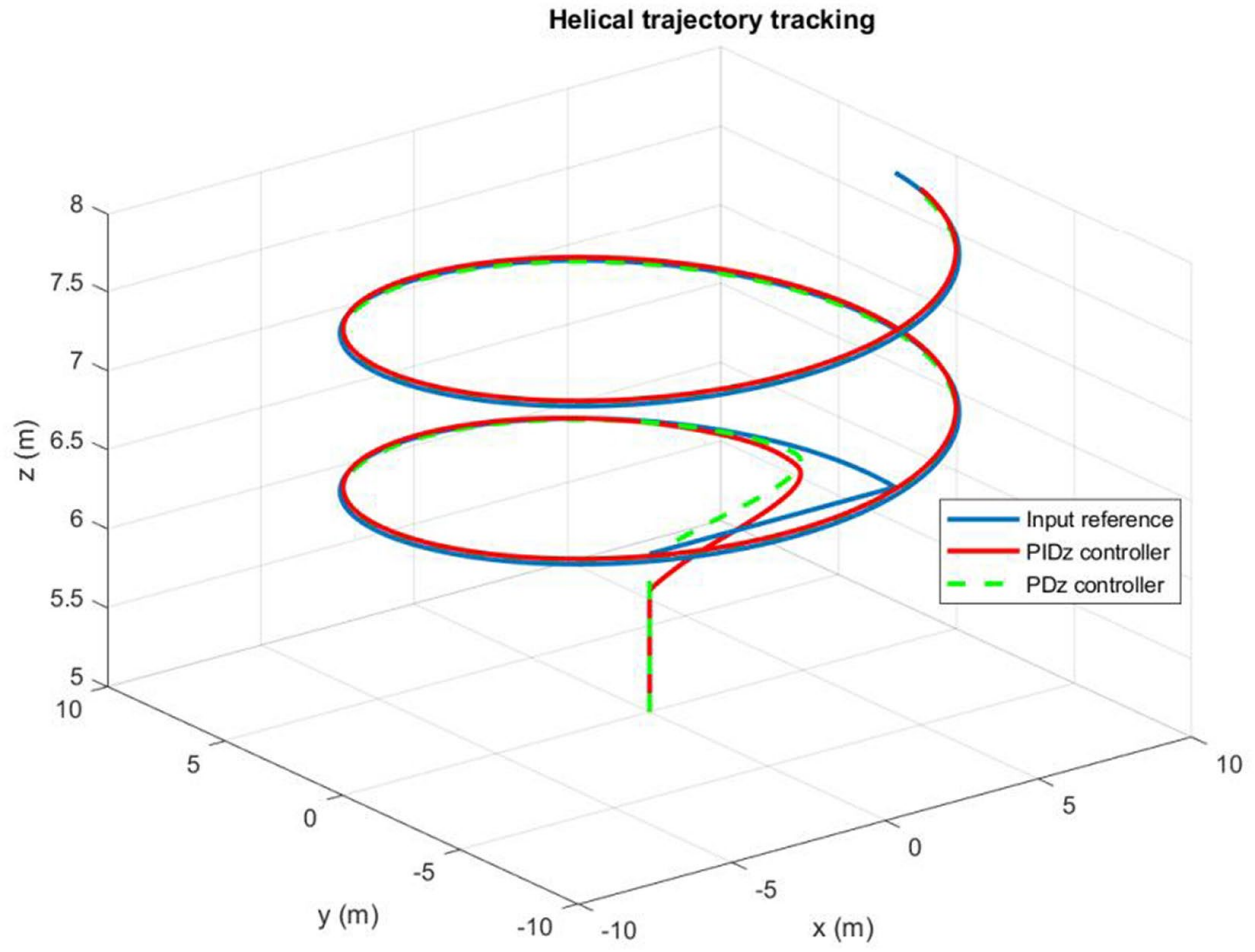
Quadrotor’s trajectory tracking.

**Figure 12:**
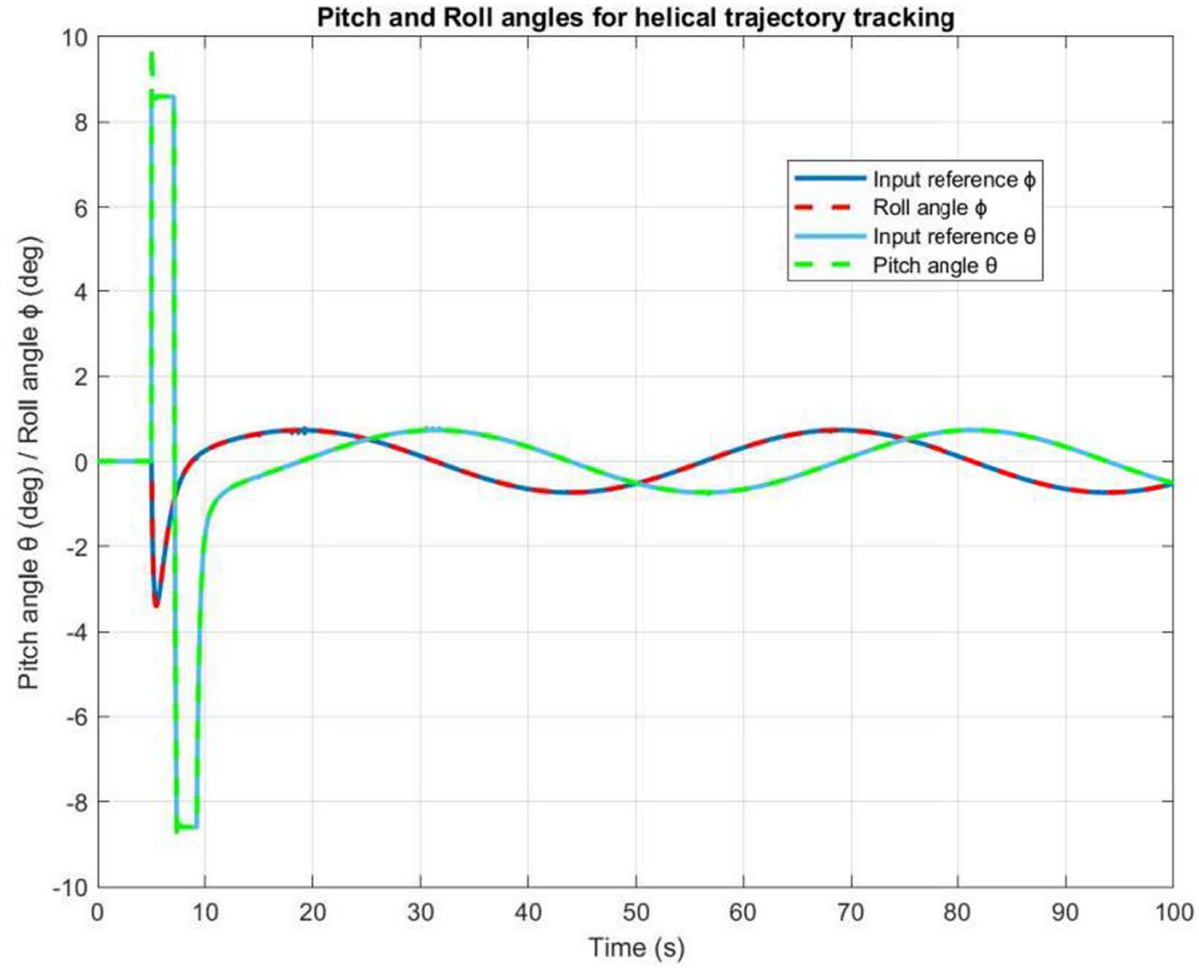
The roll and pitch angles time evolution.

Figure 11 illustrates the time history of helical paths. When the quadrotor is being controlled by a PD command, the PD controller produces a small permanent position error. However, the PID controller operates dynamically to ensure precise control. Besides, Figure 12 highlights that the pitch and roll controllers ensure that the quadrotor remains very stable. It can be observed that the trajectory tracking performance is satisfactory with both PD and PIC controllers, particularly when considering the application of the designed autopilot.

### 4.4 Assessment of the control robustness

**Figure 13:**
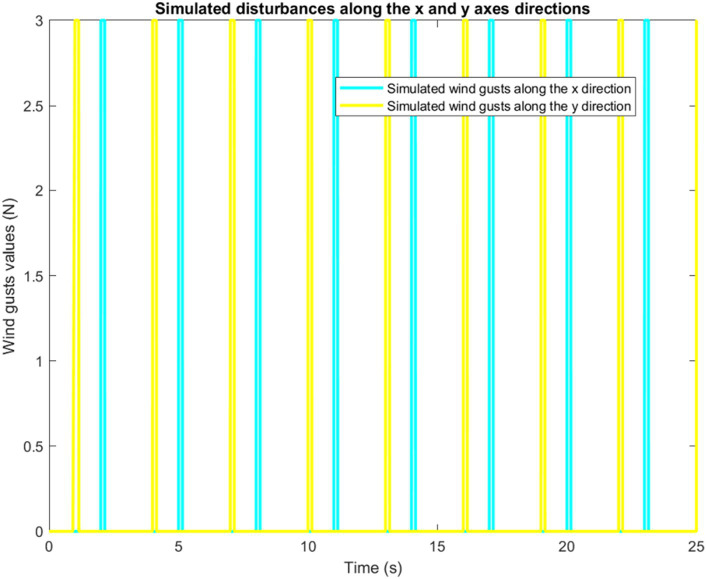
Simulation of disturbances along X and Y axes.

As an input signal to the quadrotor model, a wind force model was added to test whether the control response of the quadrotor was affected by wind disturbances. We have implement the wind force inspired from the work of Alexis et al. (2012). We begin by assessing the impact of disturbances along X and Y axes. The corresponding simulation results are reported in Figure 13.

**Figure 14:**
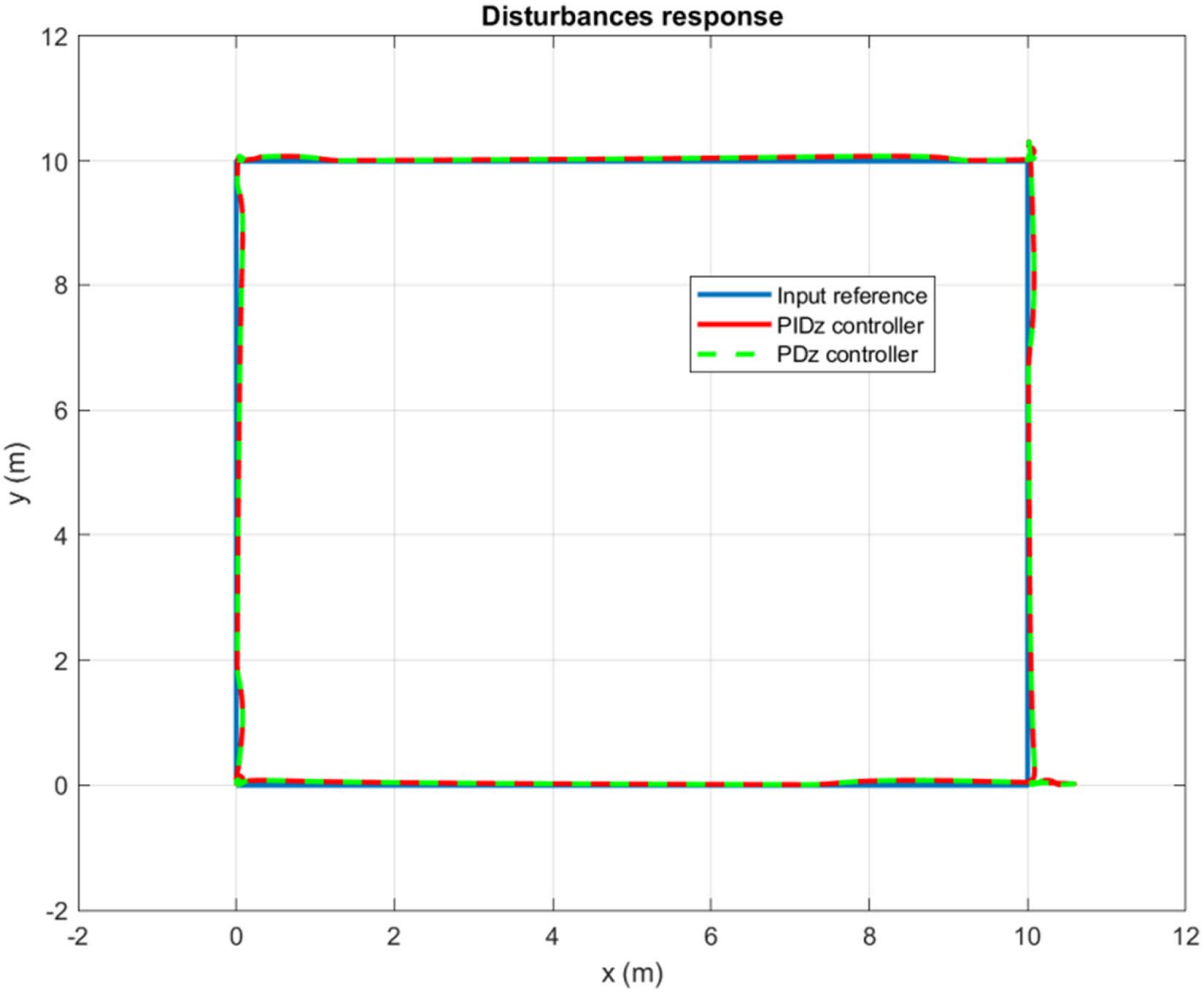
Wind gusts response for a square trajectory tracking.

It is worth mentioning that the quadrotor is capable of following any rectangular trajectory accurately in the absence of disturbances. Now, when adding the wind gusts scenario, the controller shows very good robustness. Indeed, by properly compensating for wind gusts, the controller corrects any deviation caused by the wind as displayed by Figure 14.

**Figure 15:**
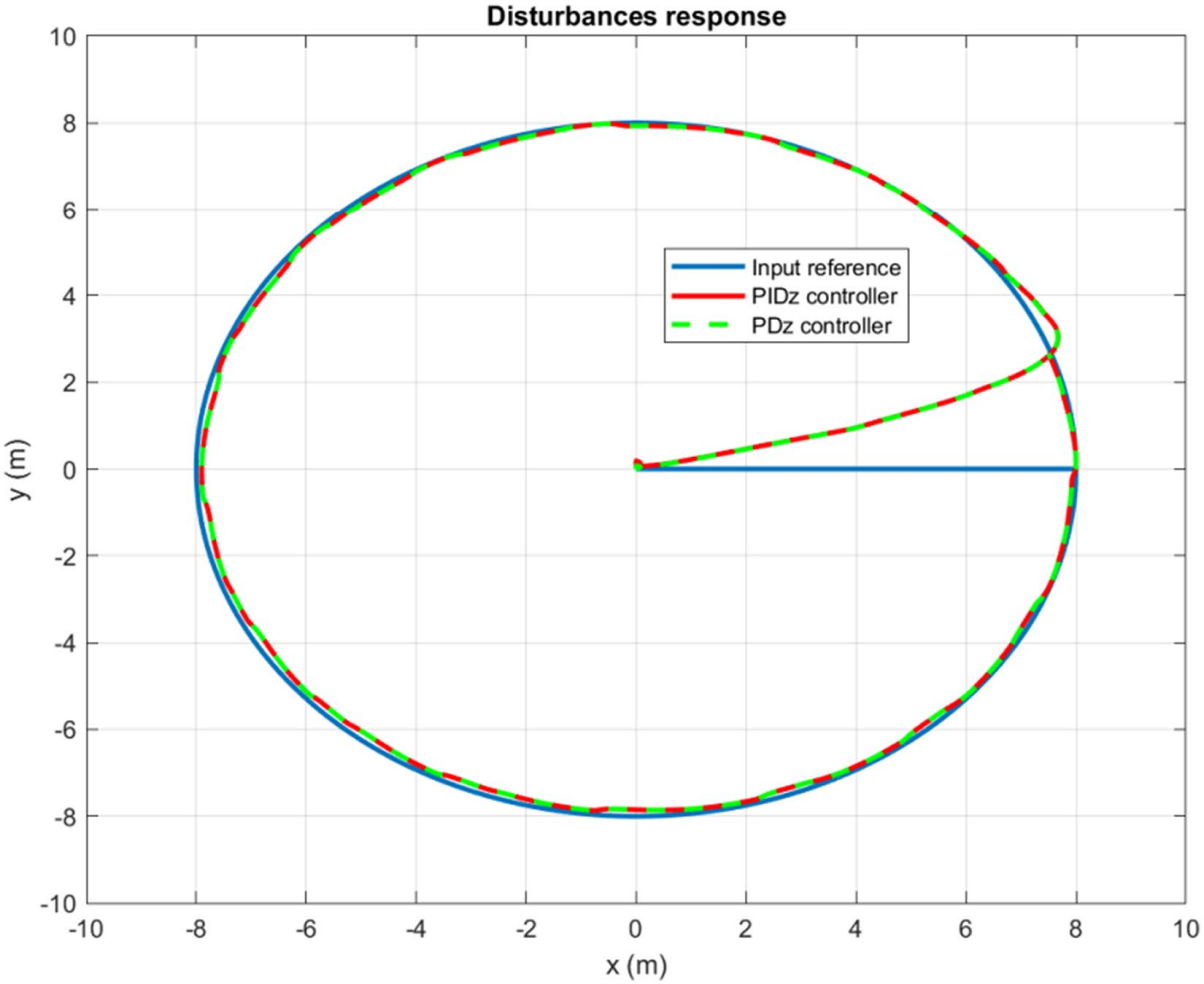
Wind gusts response for a circular trajectory tracking.

Furthermore, controlling a circular trajectory yields the same promising results. As can be clearly observed in Figure 15, the controller succeeds to keep the quadrotor’s trajectory around the input reference in a highly accurate and dynamic manner. For both circular and rectangular trajectories, the simulation results confirm that the quadrotor, utilizing the designed autopilot, can deal with disturbances equivalent to more than 30% of its weight without experiencing any significant deviation from its reference trajectory.

At this stage, it is noteworthy that as the parameters of the controllers are adjustable, their determination could be calculated in several ways and using multiple methods. The mathematical approach used in this paper consists of calculating the parameters according to a second order model that corresponds to the system response. In future work, another approach to be considered involves implementing a more intelligent strategy. This entails using machine learning algorithms trained with historical data. In fact, additional IoT sensors could be deployed for example to continuously update the parameters according to weight variation, flight behavior, environment/landscape nature, and weather information.

## 5 Conclusion

This study explores the design of an autopilot for a quadrotor unmanned aerial vehicle to analyze intelligent navigation algorithms for surveillance and detection purposes. Its purpose is to serve as a tool for simulating intelligent trajectory tracking algorithms, aiding in the enhancement and refinement of detection and surveillance tasks. Toward this goal, a configurable PD/PID controller is introduced. Simulation study of the controllers were conducted using Matlab Simulink. The simulation results, concerning main movements and trajectory tracking, were presented to demonstrate the controllers’ good performance. With the introduction of wind gusts, the proposed controllers effectively maintained the quadrotor’s trajectory close to multiple provided input references in an accurate manner. While the PID-controlled autopilot exhibited slightly superior performance, the hybrid version emerged as an enticing alternative, capitalizing on the simplicity of the PD control approach.

Future research could delve deeper into exploring controllers with improved efficiency, given the established understanding that the PID controller falls short of optimality and fails to meet all stability requirements for flight. In addition to studying the performance of the proposed control scheme with respect to other disturbances types, it would also be possible to investigate the accuracy of the quadrotor parameters as well.

## Data availability statement

The original contributions presented in the study are included in the article/supplementary material, further inquiries can be directed to the corresponding author.

## Author contributions

AB: Conceptualization, Data curation, Formal analysis, Investigation, Methodology, Software, Validation, Writing – original draft. MS: Supervision, Validation, Writing – review & editing.
